# Editorial: Immune checkpoint inhibitors in cancer: balancing the benefits with the side effects?

**DOI:** 10.3389/fendo.2023.1268402

**Published:** 2023-09-11

**Authors:** Cristiane J. Gomes-Lima, Wen Zhou, Zoe Quandt

**Affiliations:** ^1^ Department of Medicine, Oncoclinicas Group, Brasilia, DF, Brazil; ^2^ Department of Comprehensive Care, Case Western Reserve University School of Dental Medicine, Cleveland, OH, United States; ^3^ Division of Endocrinology and Metabolism, Department of Medicine, University of California, San Francisco, San Francisco, CA, United States

**Keywords:** immune checkpoint inhibitors (ICI), immunotherapy, cancer, adverse events, endocrine glands

Immune checkpoint inhibitors (ICIs) have emerged as a revolutionary treatment for multiple solid and hematologic malignancies, associated with durable treatment responses (1, 2). ICIs enhance the immune response against cancer cells by binding to checkpoints that would normally induce immune suppression, thereby allowing for immune activation. These mechanisms are a vital “brake” on the system, for example inducing immune tolerance to prevent autoimmune disease or dampening inflammation in chronic viral infections. Malignant cells will often co-opt these systems to prevent immune destruction. It is therefore not unexpected that the flip side of this process is the development of immune-related adverse events (irAEs). These irAEs occur to a varying degree in almost every organ, including the endocrine glands (3, 4). Endocrine irAEs are relatively unique, in that they are almost always permanent and persist past therapy. Furthermore, there have been multiple studies linking endocrine irAEs in particular to response to ICIs (5–7). To highlight the endocrine consequences of these treatments, Frontiers in Endocrinology organized a Research Topic related to ICIs in the treatment of endocrine cancers or other types of cancer, endocrine irAEs, and how to manage them.

For several months we received articles from many authors and from several parts of the world. The selected articles are presented here as one case report, three original research studies, two systematic reviews, and one review.

Cardona et al. provide an excellent clinical review of what is known about endocrine irAEs. They detail the epidemiology, pathophysiology, diagnosis, and management of thyroid dysfunction, pituitary dysfunction, adrenal dysfunction, diabetes, and hypoparathyroidism. They conclude with a call to action for a more in-depth study of the many facets of these events, ranging from improving clinical diagnostics and treatment to defining mechanisms of disease.

To comprehensively evaluate several metabolic and nutritional disorders (MNDs) in patients receiving ICIs, Zhai et al. analyze data from the US FDA’s Adverse Event Reporting System (FAERS) database. MNDs correlated significantly with the use of ICIs compared to all patients in the database. Notably, a significantly higher incidence of MNDs was observed in patients receiving combination therapy (CTLA-4 inhibitor with PD-1 inhibitor) rather than monotherapy. However, there were also substantial differences in the incidence of MNDs among the various forms of monotherapies (inhibitors of PD-1, PD-L1, or CTLA-4), with an especially high incidence in PD-1 inhibitor monotherapy. The study details the occurrence of MNDs during ICI therapy and systematically analyzes the significant risks related to various ICI across the US population. This information could influence the therapy choice and spotlights the importance of surveillance of endocrine function to avoid serious complications.

In another collaboration, Iwamoto et al. evaluate the incidence of endocrine irAEs in patients who were treated with ICIs in Japan. The irAEs observed include hypothyroidism, and, to a lesser extent, hypoadrenocorticism, hypopituitarism, and insulin-deficient diabetes mellitus. A significantly higher irAE incidence was observed in patients receiving combination therapy of CTLA-4 and PD-1 inhibitors rather than monotherapy. The authors correctly discuss the limits of this study including its single-center retrospective study design. This report again sheds light on the importance of periodic monitoring of endocrine function during ICI therapy to ensure better outcomes.

A systematic review by Jacques et al. focuses on pituitary irAEs including hypophysitis and hypopituitarism in cancer patients on ICI. The final analysis included 239 studies, comprising a total of 30,014 patients, in which 963 cases of hypophysitis and 128 cases of hypopituitarism were identified. In accordance with the literature, the authors find that pituitary changes were most common following ipilimumab, a CTLA-4 inhibitor, especially when in combination with PD-1 inhibitors. Patients with hypophysitis presented mostly with fatigue and headache. The main image findings were enlargement of the pituitary gland and enhanced contrast uptake, while the main hormonal changes were adrenal insufficiency followed by hypothyroidism. This study represents the largest compilation of cases of pituitary dysfunction to date and contributes to the characterization of this serious endocrine irAE.

Next, the case report by Katakura et al. describes a man with malignant melanoma who was treated with ICIs and developed thyroiditis, hypophysitis with isolated ACTH deficiency, and aseptic meningitis. This case report illustrates the variety of irAEs that may occur with the use of ICIs and emphasizes the need for clinical attentiveness for multiple simultaneous irAE.

Liang et al. complete a systematic review of the use of ICIs in triple-negative breast cancer, focusing on irAEs and response across multiple treatment lines using a random-effects model of Bayesian network meta-analysis. This study provides insight into balancing the risks and benefits of treatment in patients with this aggressive form of breast cancer. Thyroid irAEs were the most frequent form of irAEs, which is clinically important to endocrinologists as patients can receive ICIs in early-stage disease for triple-negative breast cancer and therefore have many years, conceivably even some fertile years, to be followed.

Finally, pivoting from the broader scope of endocrine irAEs, in their original research, Luo et al. address the immunological landscape of ICIs in multiple forms of advanced thyroid cancer, an area where their value has yet to be established, through expression profiles from a tissue microarray of nine immune checkpoints and then comparing this to survival. Expression of particular checkpoints varied by cancer tissue type, and at times were linked with prognosis. This article may serve as an excellent basis for the development of novel ICIs that might be more effective for advanced TCs in the future, such as VISTA or B7H3 which were expressed in papillary thyroid cancer and poorly differentiated thyroid cancer, compared to PD-L1 which was seen in anaplastic thyroid cancer and linked to response.

In summary, this Research Topic shows that there is a multitude of interactions between the endocrine organs and malignancy, both as direct targets for the immune system (e.g., thyroid cancer) and as bystanders (irAEs). As work in immunotherapy continues, it is critical to leverage insights from each field to be able to further work in cancer treatment and autoimmune disease to better treat and care for patients.

## Author contributions

CG-L: Writing – original draft, Writing – review & editing. WZ: Writing – original draft, Writing – review & editing. ZQ: Writing – original draft, Writing – review & editing.
